# Length of stay and determinants of early discharge and extended stay after cesarean delivery in a resource-limited setting: southern Ethiopia, 2023

**DOI:** 10.3389/fgwh.2024.1346720

**Published:** 2024-11-15

**Authors:** Gemeda Wakgari Kitil, Gebremaryam Temesgen Birgoda, Agmasie Damtew Walle, Dagne Deresa Dinagde, Gizu Tola Feyisa, Yosef Alemayehu Gebrehiwot, Bekem Dibaba Degefa

**Affiliations:** ^1^ Departments of Midwifery, College of Health Sciences, Mattu University, Mattu, Ethiopia; ^2^ Departments of Midwifery, College of Medicine and Health Sciences, Arba Minch University, Arba Minch, Ethiopia; ^3^ Departments of Health Informatics, College of Health Sciences, Mattu University, Mattu, Ethiopia

**Keywords:** Arba Minch Town, cesarean delivery, health facility, length of hospital stay, southern Ethiopia

## Abstract

**Background:**

For mothers and newborns to obtain the recommended postnatal care follow-up and package, the healthcare facility has to provide a minimum length of stay following delivery. Early discharge may result in a shortened recovery time, less access to resources and support, and a prolonged stay, resulting in a greater risk of postpartum depression and financial constraints. In Ethiopia, there has been no study conducted on the length of hospital stays following a cesarean delivery. Therefore, this study aimed to determine the average length of stay and identify factors influencing both early discharges and prolonged stays after cesarean delivery in southern Ethiopia.

**Methods:**

A facility-based cross-sectional study was conducted between November 23, 2022, and March 23, 2023. A systematic sampling method was used to select 367 participants, and data were collected using the Kobo Toolbox mobile application. The mean length of stay was calculated in hours, and descriptive statistics were used to summarize the data. Multinomial logistic regression was employed to analyze the determinants of length of stay, with significance set at a *p*-value of <0.05.

**Results:**

The mean duration of the length of stay of mothers in health facilities after cesarean delivery is 65 h or approximately 2.71 days (SD ± 0.77). Determinants of shorter stay included women aged 20–24 years [AOR = 5.19; 95%CI 1.51–8.23], distance from hospital 30–60 min [AOR = 2.51; 95% CI 1.12–5.73], first antenatal booking [AOR = 0.16; 95%CI 0.05–0.25], monthly income <2,000 birr [AOR = 3.11; 95%CI 1.18–6.05], had health insurance [AOR = 0.35; 95% CI 0.26–0.37] and had counseled [AOR = 0.09; 95%CI 0.07–0.154]. Extended stays were associated with severe pre-eclampsia [AOR = 2.80; 95%CI 2.41–3.27], multiple births [AOR = 2.51; 95%CI 1.34–4.71], and postoperative complications [AOR = 3.52; 95%CI 1.35–5.01].

**Conclusion:**

The average post-cesarean hospital stay is 2.71 days, with duration influenced by factors such as age, distance to the hospital, access to antenatal care, income, insurance, and the presence of complications. Targeted interventions, such as improving access to antenatal care, providing financial support, and proactively managing complications, can improve outcomes.

## 1 Introduction

Cesarean delivery is an obstetric surgical technique in which the woman's abdomen or uterus is incised to deliver her baby and effectively reduces maternal and neonatal mortality when carried out for medically indicated reasons (1, 2). Despite the steady rise in Cesarean rates around the world over the past few decades, there have been no appreciable maternal or perinatal benefits following this trend. On the other hand, available data has shown that higher Cesarean rates are associated with greater rates of maternal and neonatal morbidity when the procedure is performed without medical necessity (2–4). The increasing rates of Cesarean in several countries are pushing healthcare organizations to tackle modifiable factors to reduce not only the number of unnecessary Cesarean and related adverse health outcomes but also the prolonged length of stay (LOS) post-Cesarean (5–7).

The length of hospital stay is the hospitalization time of a woman immediately after delivery, calculated by subtracting the date of birth from the date of discharge from Cesarean (8). The duration of hospital stays following a cesarean can vary depending on several factors, including the mother's health, the baby's health, and the type of hospital where the procedure is performed. The American College of Obstetricians and Gynecologists (ACOG), and the American Academy of Pediatrics (AAP), recommend a hospitalization period of at least 72–96 h, while the Ethiopian Ministry of Health (MOH) suggests a minimum of 72 h following cesarean deliveries. This recommendation is based on the understanding that this is the most critical period for ensuring maternal and neonatal health (9, 10).^1^

A major public health problem arises from the increasing rate of Cesarean (11). This is because, when compared to vaginal delivery, Cesarean delivery has been linked to both short- and long-term health consequences for women, their offspring, and their households (11). Inadequate time to provide support and guidance to women in healthcare facilities can have several negative consequences. It may lead to decreased self-confidence among mothers and result in issues related to breastfeeding, maternal depression, or dissatisfaction with the care they receive (12, 13). Additionally, prolonged hospital stays can bring about various problems, including a higher risk of healthcare-associated infections, limited access to care for those who require it, unnecessary healthcare expenses, sleep disturbances, reduced support for infant feeding, and increased exposure to unfavorable conditions within the facility (12–14). These factors reduce the mother's trust, the father's involvement, or family bonds (13). Moreover, both extended hospital stays and premature discharges that result in re-admissions are inefficient and contribute to increased economic costs for both families and the healthcare system (15, 16).

The decline in LOS is steadily increasing the demand for postnatal services, both in quantity and quality, worldwide (16, 17). Nowadays, in Ethiopia, especially in the era of the COVID-19 pandemic, early hospital discharge has become a widespread practice (18). These stays can lead to health problems, dissatisfaction, or increased costs. Especially for short stays, there may not be enough time left to identify, diagnose, or treat complications, which may result in increased morbidity and mortality (17, 19, 20). Also, in a Turkish study, insomnia, malaise, and constipation were the most prevalent health problems for women after they were discharged early (21). Similarly, in the United Kingdom, the length of stay after childbirth is steadily decreasing, owing to cost-saving, which is one of the reasons for early discharge (22).

While institutional deliveries increased more rapidly than home deliveries, the length of hospital stay (LOS) after cesarean has decreased more significantly compared to vaginal delivery in the United States (USA), dropping by 53.8% from 3.6 days in 2006 to 2–3 days in 2020 (23, 24). A Danish study conducted from 2001 to 2014 reported a median LOS reduction from approximately 4.7 days to 2.7 days after Cesarean (25). According to a previous study conducted in 30 low- and middle-income countries, the average length of stay after cesarean delivery in health facilities ranged from 2.5 to 9.3 days (23).

The Sustainable Development Goal Target 3.1 aims to reduce the global maternal mortality ratio to less than 70 per 100,000 live births by 2030. Understanding the factors influencing the length of hospital stay after cesarean delivery is essential for achieving SDG Target 3.1 and improving maternal health outcomes, as appropriate postnatal care is indicated by the duration of hospitalization (10, 13).

Hospital length of stay serves as an indicator of hospital activity and resource efficiency. The variability in post-Cesarean section stay duration can be attributed to factors such as practice patterns, service efficiency, and patient preferences (23, 26, 27). A woman's postpartum stay depends on factors including facility type, mode of delivery, obstetric complications, and socioeconomic factors (23, 26). Research shows that older age, those with higher socioeconomic status and education, and those with private insurance tend to have longer stays. Conversely, early discharge is associated with younger age, lower socioeconomic status, multiparity, preexisting medical conditions, previous cesarean deliveries, inadequate discharge planning, and infant-related issues like fetal anomalies, macrosomia, and restricted intrauterine growth (23, 26, 28). In Nepal, research suggests that physician-assisted delivery, lower parity, lower economic status, and low birth weight lead to longer stays (25). In India, factors associated with shorter stays include late initiation of prenatal care, public hospital delivery, family decisions for early discharge, and lack of insistence by health workers for a longer stay (29).

Understanding the factors influencing the duration of hospital stays for women undergoing cesarean delivery is crucial for enhancing postpartum care and improving maternal and neonatal health outcomes. However, there has been no study conducted on the length of hospital stays and their determinants in Ethiopia. Therefore, this study aims to investigate the duration of post-childbirth hospital stays and the factors influencing them in Southern Ethiopia.

## 2 Methods and materials

### 2.1 Study area and period

This study was conducted in Arba Minch Town Public Hospital, which is located 504 km south of Addis Ababa, the capital city of Ethiopia, from November 23, 2022, to March 23, 2023. In the town, there are two public hospitals; Arba Minch General Hospital and Dilfana Primary Hospital. Arba Minch General Hospital was built in 1961 to serve around 500,000 people, but now it is serving more than two million people. Over 100,000 patients visit the hospital every year.^2^ Dilfana Primary Hospital was upgraded from Sikela Health Center in 2021. In general, the hospital provides preventives, curatives, and rehabilitative services for the Gamo zone and nearby zones.

### 2.2 Study design

An institutional-based cross-sectional study design was applied.

### 2.3 Study population

A woman who underwent a cesarean at Arba Minch General Hospital and Dilfana Primary Hospital during data collection.

### 2.4 Inclusion criteria

All women who underwent cesarean during the study period.

### 2.5 Exclusion criteria

Women who were diagnosed with obstructed labor (because they stayed in health facilities for more than 7 days).

### 2.6 Sample size determination

The required sample size was determined by using the single population proportion formula with the following assumption; 33% of women stay less than 72 h (early discharge) in Hospital after post-Cesarean according to the 2016 EMONC report,^2^ 95% confidence interval, 5% marginal error and 10% for non-response rate.

*N* = [*Z*^2^ *p*(1-p)^2^]/*d*^2^ = 340 by adding 10% of the non-response rate the final sample size was 374.

### 2.7 Sampling technique and procedure

During the data collection phase, a systematic random sampling technique was employed to select participants. The total number of women who underwent cesarean at both hospitals was determined by referencing the client registration book or records from the previous year for the corresponding months at both institutions. In 2021, Arba Minch General Hospital recorded 350 cesarean deliveries, while Dilfana Primary Hospital recorded 120, resulting in a combined total of 470 cesarean deliveries across both facilities. Utilizing this information as the sampling frame, the sample size for the study was calculated, resulting in a total sample size of 374. Proportions-to-size allocations were then assigned to both hospitals before the selection of study participants. Consequently, 279 women were selected from Arba Minch General Hospital, and 95 women were selected from Dilfana Primary Hospital. All eligible women were chosen at every k^th^ interval.

### 2.8 Outcome variables

**Length of hospital stay** (early, appropriate, and late discharge), which was computed using the difference between the date of discharge and the date of the Cesarean procedure done.

### 2.9 Determinants of LOS

### 2.10 Socio-demographic factors

The age of the woman at the time of childbirth was categorized into five groups (<20, 20–24, 25–29, 30–34, and >35 years). Marital status was defined as married or unmarried. Educational status was categorized as follows: unable to read and write, able to read and write, primary education, secondary and college, and above. Family monthly income was categorized as <2,000 ETB, 2,000–4,000 ETB, and more than 4,000 ETB. Distance from the hospital was classified as <30 min, 30–60 min, and >60 min. Companionship by a family member was categorized as husband, mother, sister, or brother.

### 2.11 Obstetric related factors

Parity was defined as primigravida or multigravida. Pregnancy complications included conditions like pregnancy-induced hypertension (PIH), premature rupture of membranes (PROM), antepartum hemorrhage (APH), and gestational diabetes. Age at the first pregnancy was categorized into three groups (<20, 21–25, and >26).

Antenatal care (ANC) follow-up was defined by the presence or absence of visits. Delivery attendants could be integrated emergency obstetric surgery, clinical midwives, or gynecologists. The frequency of Cesarean delivery), including primary or secondary Cesarean.

**Facility-related factors** the setting of the delivery, whether in a general or primary hospital.

**Fetal-related Factors**: Newborn birth weight was recorded in grams from the maternal card. Gestational age at birth was categorized as 28–36 weeks, 37–39 weeks, and 40 weeks and above.

### 2.12 Conceptual framework

**Figure 1:**
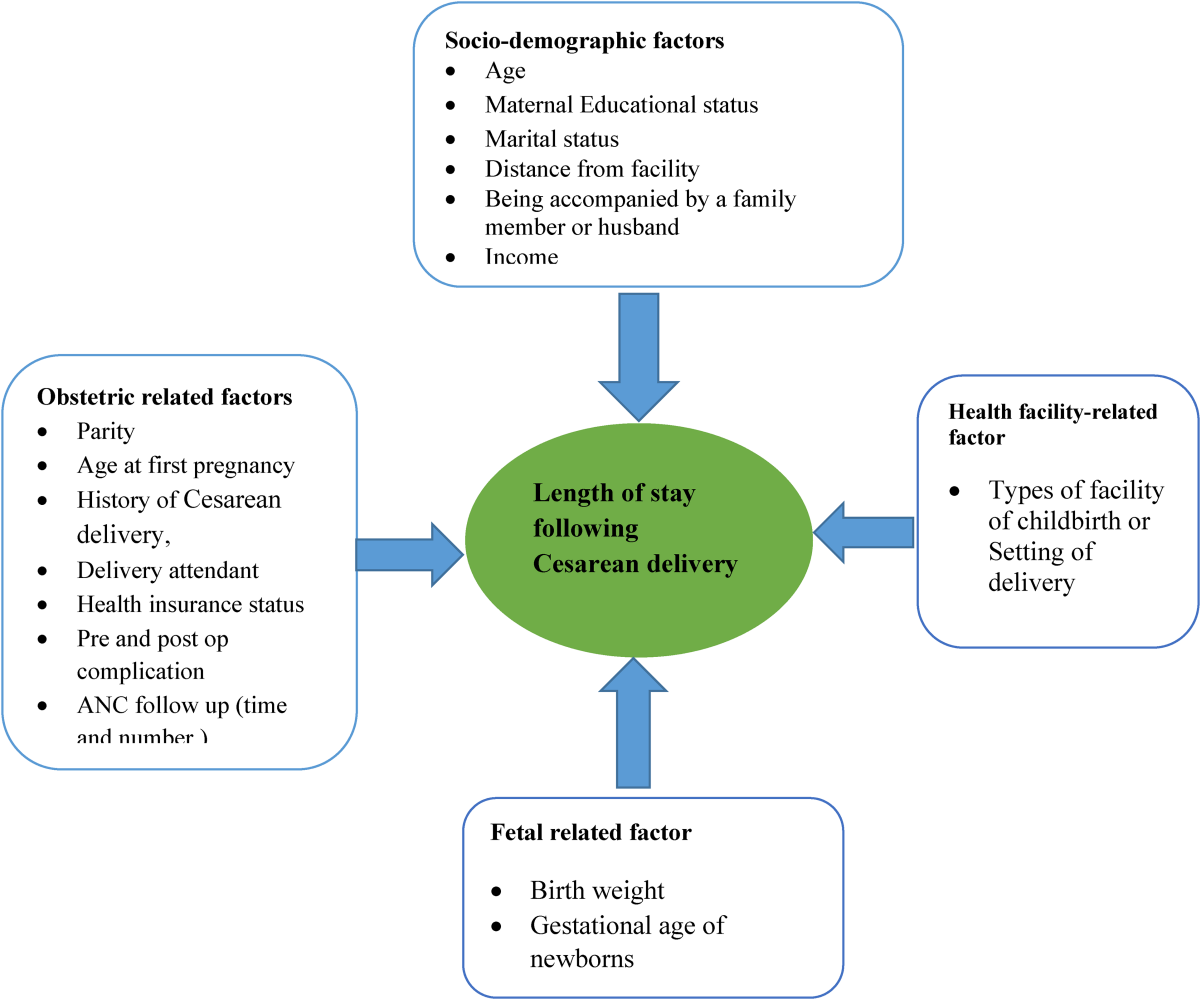
Conceptual framework of length of hospital stay and its determinants adapted from different kinds of literature (30, 31).

Some studies have found that some factors determine the length of hospital stay after Cesarean and this conceptual framework was developed/adopted after reviewing different kinds of literature and the Ethiopian Emergency Obstetric and Newborn Care (EmONC) 2016 report (Figure 1).

### 2.13 Operational definitions

**Length of hospital stay (LOS)** is the number of days the women stayed in the hospital from the date Cesarean was done until the women were discharged from the hospital (i.e., the time from the date Cesarean was done to the time at which they were discharged was calculated in an hour and then extracted into days. For example, LOS one day is converted to 24 h) (8).

**Appropriate hospital stay or discharge:** discharge of a mother from the hospital after completion of 72 to 96 h of childbirth (4, 10).

**Early hospital discharge:** when the women who were given birth through Cesarean were discharged before 72 h of their hospital stay.

**Late discharge (from hospital):** when women who were given birth through Cesarean were not discharged after 96 h of hospital stay**.**

**Post-Cesarean complication:** Those women who develop at least one of the following after Cesarean has been done: wound infection, postoperative fever, anemia, distended abdomen, postpartum blood pressure elevation, and admission to the NICU, and prematurity were the most common newborn complications, respectively (3, 4).

### 2.14 Data collection procedures

Data collection involved the use of a structured and pre-tested questionnaire, which was adapted from relevant studies and the Ethiopian Emergency Obstetric and Newborn Care Assessment Report of 2016 (30). The questionnaire was initially prepared in English, translated into Amharic, and then back into English to ensure consistency. The data was gathered by three midwives with BSc degrees who were chosen from different healthcare facilities. The data collection process was supervised by a BSc midwife in a selected healthcare facility and the principal investigator.

The collected data encompassed socio-demographic, obstetric, and fetal-related characteristics, which were extracted from maternal records and referral notes. Additionally, face-to-face interviews were conducted on discharge days to ascertain the date of the cesarean procedure and the discharge date, allowing for the calculation of hospital stay duration.

### 2.15 Data quality assurance

To ensure data quality, several measures were implemented. Data collectors received training and were supervised appropriately, with overall supervision carried out by the principal investigator. Before the start of the data collection, the questionnaire was pre-tested on 19 women who had cesarean deliveries at Chencha Primary Hospital. This allowed us to assess the validity of the instrument and the respondents’ responses to the questions. Based on the results of the pretest, we made necessary adjustments to the questions before the actual data collection commenced. The maintenance of privacy and confidentiality of the respondents, as well as effective communication skills between respondents and interviewers that were gained through training sessions for both data collectors and supervisors, contributed to the quality of the study.

Additionally, to maintain data accuracy, we conducted daily reviews of all questionnaires at the end of each data collection day. Any errors, such as double data entry, missing values, inconsistencies, or outliers, were promptly identified and corrected by the supervisor and data collector.

### 2.16 Data processing and analysis

The data was collected using the Kobo toolbox mobile application and then transferred to Statistical Package for Social Science (SPSS) version 26.0 for analysis. To ensure data quality, missing values, and inconsistencies were identified by running frequency checks and other exploratory procedures. Descriptive statistics, such as frequency distributions, mean, and median, were computed. Multinomial logistic regression analysis was employed to examine the relationship between dependent and independent variables. In the multinomial logistic regression analysis, “appropriate hospital stay/discharge on appropriate days” served as the reference category for the dependent variable and was compared with the other categories, including early hospital discharge vs. discharge on appropriate days and late hospital discharge vs. discharge on appropriate days. To identify significant and independent factors associated with the Length of Hospital Stay, all clinically relevant variables and those showing at least a marginal association (*p* < 0.25) in the bivariate analysis were included in the multinomial logistic regression analysis of LOS.

In the result and discussion section, both the crude and adjusted odds ratios with 95% confidence intervals were calculated and presented. A *p*-value less than 0.05 was considered statistically significant. The chi-square statistic was used to assess whether the change in unexplained variance from the baseline model to the final model was significant. The final model was tested using the Pearson and deviance statistics, resulting in *P*-values of 0.983 and 1.000, respectively.

## 3 Results

### 3.1 Socio-demographic characteristics of participants

Out of a total of 374 women who underwent a cesarean, 367 provided data, resulting in a response rate of 98.1% (7 did not respond as they were in a hurry to leave). The mean age of the study participants was 28.2 (±5.2 SD) years. Around 352 (96%) of the women were married, making up about two-thirds of the ethnically Gamo 246 (67%) women. Additionally, 246 (67%) of the women identified themselves as housewives, and 139 (37.9%) had no formal education. About 165 (45.0%) of the women had to travel 30–60 min to reach the hospital (Table 1).

**Table 1:** Socio-demographic characteristics of the women who gave birth through cesarean delivery at Arba Minch general hospital and Dilfana primary hospital, 2023 (*n* = 367).

Variables	Category	Frequency (n)	Percent (%)
Age in years	<20	22	6.0%
	20–24	69	18.8%
	25–29	141	38.4%
	30–34	81	22.1%
	>35	54	14.7%
Religion	Orthodox	174	47.4%
	Protestant	155	42.2%
	Muslim	38	10.4%
Residence	Urban area	172	46.9%
	Rural area	195	53.1%
Ethnicity	Gamo	246	67.0%
	Gofa	26	7.1%
	Sidama	33	9.0%
	Wolaita	44	12.0%
	Others	18	4.9%
Educational level	Not read and write	139	37.9%
	Able to read and write	48	13.1%
	Primary education	99	27.0%
	Secondary	37	10.1%
	College and above	44	12.0%
Occupation of mother	Gov.t Employee	58	15.8%
	Housewife	246	67.0%
	Private	19	5.2%
	Merchants	44	12.0%
Marital status	Married	352	96.0%
	Unmarried^a^	15	4.0%
Family monthly income	≤2,000 ETB	88	24%
	2,000–4,000 ETB	127	34.6%
	≥4,000 ETB	152	41.4%
Distance from hospital	<30 min	69	18.8%
	30–60 min	165	45.0%
	≥60 min	133	36.2%

^a^ Unmarried, widowed, divorced, and single; ETB, Ethiopian birr.

### 3.2 Obstetric characteristics of women

About 183 (49.9%) of the women experienced their first pregnancy between the ages of 21 and 25. Almost all of the participants, 357 (97.3%), received antenatal care (ANC). Among these, 83.2% of the women had four or more ANC visits. Approximately one-fourth of the women (24.8%, 91) experienced pregnancy-related complications. Among these complications, pregnancy-induced hypertension was the most common, accounting for 43.9%. One in eleven women (9.3%, 34) experienced multiple types of pregnancy-related issues. Nearly 55 women (15%) gave birth before 37 weeks of pregnancy. Approximately 73% (268) of the participants underwent emergency cesarean deliveries. The most common indication for Cesarean was non-reassuring fetal heart rate patterns (40.1%). Around 22.9% (84) of the women experienced postoperative complications, with post-op fever (31%) and elevated blood pressure (26.2%) being the most prevalent (Table 2).

**Table 2:** Obstetric characteristics of the women who gave birth through cesarean delivery at Arba Minch general hospital and Dilfana primary hospital, 2023 (*n* = 367).

Variables	Category	Frequency (*n)*	Percent (%)
Age at first pregnancy	<20	66	18.0%
	21–25	183	49.9%
	26–30	118	32.2%
Parity	Prim gravida	98	26.7%
	Multigravida	269	73.3%
Antenatal care follow-up for this pregnancy	Yes	357	97.3%
	No	10	2.7%
First antenatal care booking for this pregnancy (*n* = 357)	<12 weeks	109	30.5%
	13–27 weeks	155	43.4%
	>28 weeks	93	26.1%
Pregnancy-related complications during this pregnancy	Yes	91	24.8%
	No	276	75.2%
Types of pregnancy complication (*n* = 91)	Pregnancy-induced hypertension	40	43.9%
	Premature rupture of membrane	28	30.8%
	Antepartum hemorrhage	15	16.5%
	Gestational diabetes	8	8.8%
Gestational age of newborn at birth	<37 weeks	55	15.0%
	37–39 weeks	136	37.1%
	>40 weeks	176	48.0%
Indications for cesarean delivery	1 C/S and above	110	30.0%
	Mal-presentation	57	15.5%
	Severe preeclampsia	29	7.9%
	Failed induction	24	6.5%
	NRFHR patterns	147	40.1%
Frequency of cesarean delivery including the current	Primary	234	63.8%
	Secondary	133	36.2%
Surgeon	Integrated emergency		
	Obstetric surgery	196	53.4%
	Clinical midwife	57	15.5%
	Gynecologist	114	31.1%
Accompanied by	Husband	345	94.0%
	Mother	13	3.5%
	Sister and brother	9	2.5%
Health insurance status	Insured	77	21%
	Self-pay	290	79%
Post-operative complications	Yes	84	22.9%
	No	283	77.1%
Types of complications	Abdominal distension	15	17.8%
	Wound site discharge	9	10.7%
	Fever	26	31.0%
	Elevated blood pressure	22	26.2%
	Other^a^	12	14.3%
Have you counsel and advice on post-operative care before discharge	Yes	334	91.0%
	No	33	9.0%

^a^ Anemia, breast engorgement.

### 3.3 Fetal-related characteristics

From the total of 384 neonates delivered, the majority of 267 (69.5%) neonates were born with a birth weight of 2,500–4,000 g, while only 35 (9.1%) newborns were born with a low birth weight (birth weight <2,500 g). In terms of gestational age 1 in 7, 58 (15%) of them were born prematurely (Table 3).

**Table 3:** Fetal-related characteristics for women who underwent cesarean delivery at Arba Minch general hospital and Dilfana primary hospital, 2023 (*n* = 367).

Variables	Category	Frequency (*n*)	Percent (%)
Birth weight of newborn (in grams) (*n* = 384, 17 twins)	A. <2,500 g	35	9.1%
	B. 2,500–4,000 g	267	69.5%
	C. >4,000 g	82	21.4%
Gestational age of newborns at birth	<37 weeks	58	15.0%
	37–39 weeks	142	37.0%
	≥40 weeks	184	48.0%

### 3.4 Length of hospital stay

**Figure 2:**
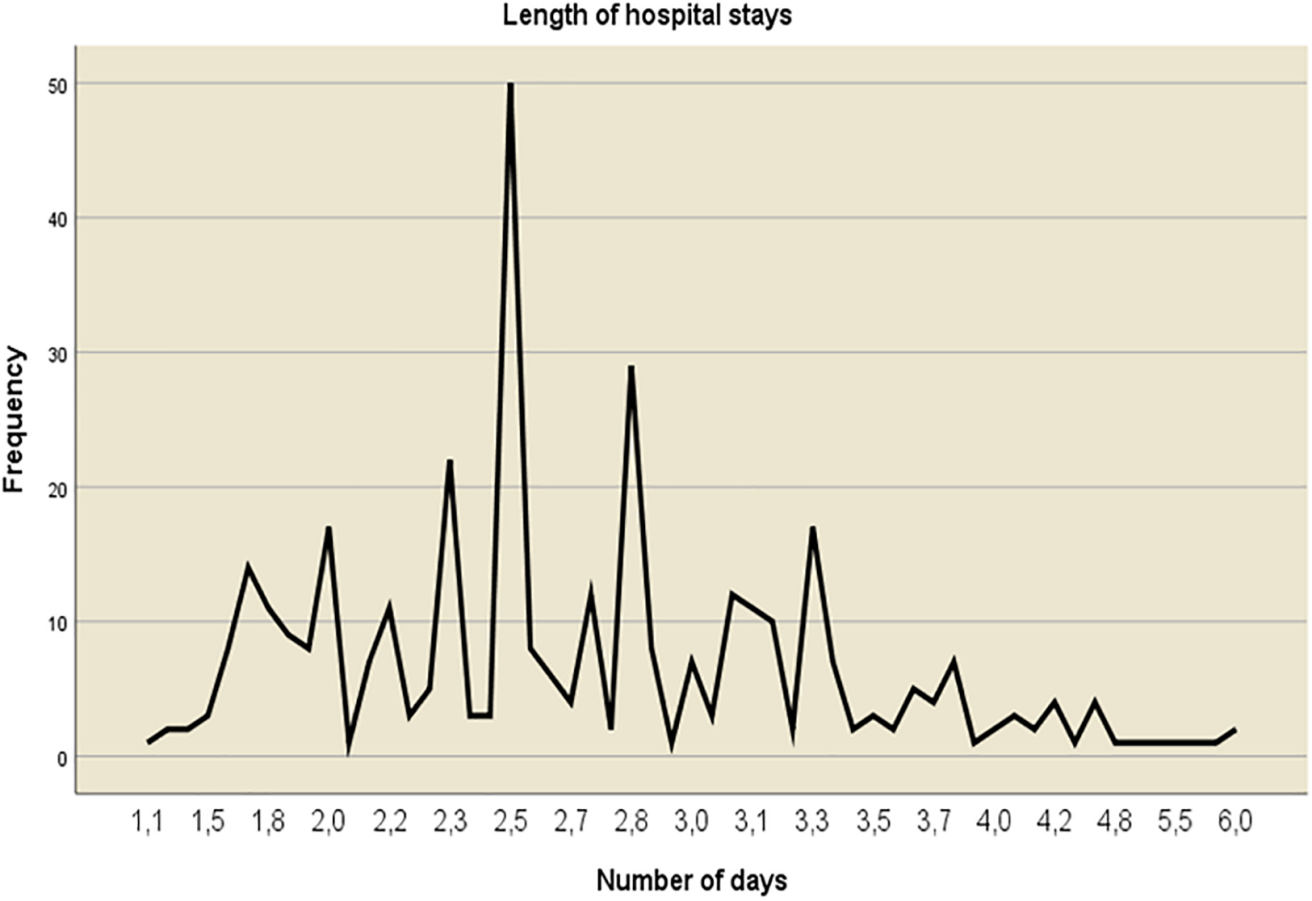
Length of hospital stay (in days) among women who underwent cesarean delivery at Arba Minch general hospital and Dilfana primary hospital, 2023 (*n* = 367).

In this study, the average duration of mothers’ stays in health facilities after cesarean delivery is 65 h or approximately 2.71 days (SD ± 0.77), with a median stay of 2.58 days (ranging from 2.17 to 3.13 days (Figure 2).

**Figure 3:**
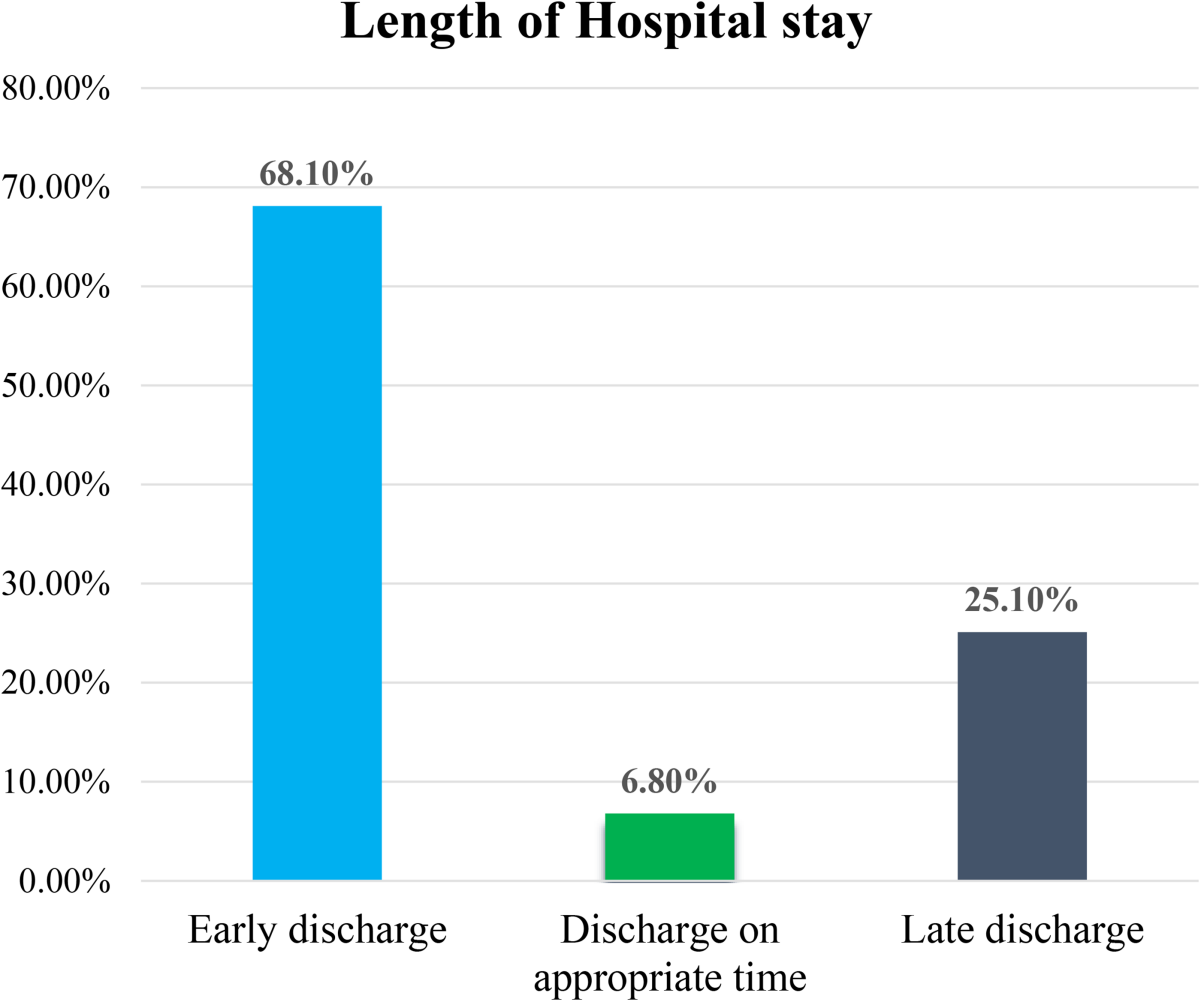
Percentage of hospital stay duration (early, appropriate, and late discharge) among women who underwent cesarean delivery at Arba Minch general hospital and Dilfana primary hospital in 2023 (*n* = 367).

Out of the total 367 women who underwent a cesarean delivery, 250 (68.1%) were discharged early, 92 (25.1%) were discharged on the appropriate days, and 25 (6.8%) were discharged late (Figure 3).

Out of the total 367 women who underwent cesarean delivery, 268 (73.0%) of them had emergency cesarean. Among these, 166 (61.9%), 80 (29.9%), and 22 (8.2%) were discharged early, at the appropriate time, and late, respectively. Additionally, out of the 99 women who had elective cesarean, 84 (84.9%), 12 (12.1%), and 3 (3.0%) were discharged early, at the appropriate time, and late, respectively.

### 3.5 Determinants of length of hospital stay

Sixteen variables were considered as candidates for multi-variable logistic regression with a *p*-value of <0.25. These variables included age, educational level, family monthly income, distance from the hospital, health insurance status, first ANC booking, types of pregnancy, gestational age and weight of the newborn at birth, types of Cesarean delivery, indications for Cesarean delivery, frequency of Cesarean delivery, pre and post-operative complications, birth attendant/surgeons, and advice and counsel before discharge.

Subsequently, multinomial logistic regression was used to identify factors associated with the length of hospital stay. Age, monthly income, distance from the hospital, health insurance status, first ANC booking, indications for Cesarean delivery, types of pregnancy, postoperative complications, and advice and counsel before discharge were found to be significantly associated variables with the length of hospital stay.

Consequently, women in the age group of 20–24 years were five times more likely to be discharged from the hospital early compared to those aged 35 years or older (AOR = 5.19, 95% CI 1.51–8.23). Women with a monthly income less than 2,000 ETB were three times more likely to be discharged early from the hospital compared to those with a monthly income greater than or equal to 4,000 ETB (AOR = 3.11, 95% CI 1.21–6.05).

Women who traveled to a hospital for 30–60 min, as compared to those who traveled for more than 60 min, were 2.5 times more likely to be discharged early from the hospital than those who were discharged at the appropriate time (AOR = 2.51; 95% CI 1.12–5.73). Women who booked their first antenatal care (ANC) within the first trimester (0–13 weeks), in contrast to those who booked their last trimester (after 28 weeks), were 84.0% less likely to experience shorter stays or early discharge from the hospital compared to those who were discharged at the appropriate time (AOR = 0.16; 95% CI 0.05–0.25). On the other hand, women who received counseling and advice about their health status and post-operative care before being discharged from the hospital, as compared to those who did not receive counseling and advice, were 91.0% less likely to be discharged early than those who were appropriately discharged (AOR = 0.09; 95% CI 0.07–0.154).

Furthermore, women who paid hospital costs with health insurance, in comparison to those who self-paid, were 65% less likely to experience shorter stays or early discharge from the hospital than those who were discharged at the appropriate time (AOR = 0.35; 95% CI 0.26–0.37) (Table 4).

**Table 4:** Multinomial logistic regression analysis of factors associated with shorter hospital stay/early discharge for women who gave birth by cesarean delivery at Arba Minch general hospital and Dilfana primary hospital in 2023 (*n* = 367).

	Length of hospital stay (early discharge vs. discharge on appropriate days)					
	Early discharge	Appropriate discharge	Late discharge	COR AOR		*P*-value
Age category						
<20	11 (50%)	9 (40.9%)	2 (9.1%)	0.65 (0.23, 1.84)	.87 (.16, 4.80)	0.009
20–24	54 (78.3%)	11 (15.9%)	4 (5.8%)	2.60 (1.10, 6.17)	5.19 (1.51, 8.23)	
25–29	88 (62.4%)	38 (27.0%)	15 (10.6%)	1.23 (0.62, 2.44)	1.10 (.42, 3.0)	
30–34	63 (77.8%)	16 (19.8%)	2 (2.5%)	2.09 (0.94, 4.60)1	1.81 (.57, 5.4)1	
>35	34 (63.0%)	18 (33.3%)	2 (3.7%)			
Monthly income						
<2,000	70 (79.5%)	15 (17.0%)	3 (3.4%)	2.40 (1.24, 4.58)	3.11 (1.21, 6.05)	0.031
2,001–4,000	81 (63.8%)	33 (26.0%)	13 (10.2%)	1.55 (.89, 2.69)	1.45 (0.68, 3.01)	
>4,000	99 (65.1%)	44 (28.9%)	9 (5.9%)	1	1	
Distance of your home from the hospital						
<30 min	53 (76.8%)	12 (17.4%)	4 (5.8%)	2.78 (1.34, 5.78)	2.66 (.85, 8.38)	0.026
30–60 min	127 (77.0%)	36 (21.8%)	2 (1.2%)	2.22 (1.31, 3.76)	2.51 (1.12, 5.73)	
≥60 min	70 (52.6%)	44 (33.1%)	19 (14.3%)	1	1	
Health insurance status						
Insured	46 (59.7%)	27 (35.1%)	4 (5.2%)	.54 (.31, .94)	.35 (0.26, 0.47)	0.009
Self-pay	204 (70.3%)	65 (22.4%)	21 (7.2%)	1	1	
First ANC booking for this pregnancy						
0–12 weeks	77 (68.8%)	29 (25.9%)	6 (5.4%)	0.50 (0.24, 1.04)	.16 (.05, .25)	0.001
13–27 weeks	104 (65.4%)	50 (31.4%)	5 (3.1%)	0.39 (0.19, 0.98)	.17 (.06, 1.44)	
28–42 weeks	69 (71.9%)	13 (13.5%)	14 (14.6%)	1	1	
Counsel and advice on post-operative care before discharge						
Yes	220 (65.9%)	90 (26.9%)	24 (7.2%)	.16 (.04, .69)	.09 (.07, .15)	0.009
No	30 (90.9%)	2 (6.1%)	1 (3.0%)	1	1	

### 3.6 Late discharge/extended stay vs. discharge on appropriate days

Women who gave birth to multiple neonates were 2.5 times more likely to experience longer hospital stays compared to women who gave birth to singleton neonates (AOR = 2.51; 95% CI 1.34, 4.71). Furthermore, women whose Cesarean was performed due to severe pre-eclampsia were 2.8 times more likely to have extended hospital stays compared to those who had the cesarean for non-reassuring fetal heart rate patterns (AOR = 2.78; 95% CI: 2.41–3.27). Finally, women who developed postoperative complications were 3.5 times more likely to have longer hospital stays than those who were discharged on appropriate days, in comparison to those who did not experience postoperative complications (AOR = 3.52; 95% CI 1.35–5.01) (Table 5).

**Table 5:** Multinomial logistic regression analysis of factors associated with prolonged hospital stay for women who gave birth by cesarean at Arba Minch general hospital and Dilfana primary hospital in 2023 (*n* = 367).

Late discharge vs. discharge on appropriate days						
	Early discharge	Appropriate discharge	late discharge	COR	AOR	*P*-value
Type of pregnancy						
Singleton	236 (70.9%)	77 (23.1%)	20 (6.0%)	1	1	
Twin or triplets	14 (41.2%)	15 (44.1%)	5 (14.7%)	1.28 (1.02, 4.46)	2.51 (1.34, 4.71)	0.031
Indication of cesarean delivery						
1 C/S and above	94 (85.5%)	15 (13.6%)	1 (0.9%)	.47 (.05, 4.20)	.027 (.00, 1.88)	0.008
Mal-presentation	35 (61.4%)	16 (28.1%)	6 (10.5%)	2.6 (.74, 9.34)	1.39 (.20, 9.9)	
Severe preeclampsia	7 (24.1%	12 (41.4%)	10 (34.5%)	5.8 (1.8, 9.3)	2.78 (2.41, 3.27)	
Failed Induction	15 (62.5%	7 (29.2%)	2 (8.3%)	2.0 (.33, 11.97)	.50 (.02, 11.3)	
NRFHR patterns	99 (67.3%	42 (28.6%)	6 (4.1%)	1	1	
Post-operative complications						
Yes	26 (31.0%)	39 (46.4%)	19 (22.6%)	4.3 (1.57, 11.78)	3.52 (1.35- 5.01)	0.001
No	224 (79.2%)	53 (18.7%)	6 (2.1%)	1	1	

Reference category: discharge on appropriate days; Model assumption (Goodness of fit—both Pearson and deviance statistic**)**: Chi-square = 527.351 *P* = .983, and 335.082, *P* = 1.000 respectively. Overall model fitting (Likelihood Ratio test): Chi-square = 241.951, *P* < 0.001. 1 Reference category of independent variables.

## 4 Discussion

In this study, the overall average duration of mothers’ stays in health facilities after cesarean delivery is 65 h or approximately 2.71 days (SD ± 0.77), with a median stay of 2.58 days (ranging from 2.17 to 3.13 days). Out of the total 367 women who underwent a cesarean delivery, 250 (68.1%) were discharged early, 92 (25.1%) were discharged on the appropriate days, and 25 (6.8%) were discharged late.

The result of this study (with a mean of 2.71 days) is consistent with a previous study conducted in eastern Sudan, which reported a mean duration of 2.7 days for hospital stays (31). Similarly, this research showed that the median length of hospital stay was 2.58 days (range: 2.17–3.13 days). This finding is consistent with a study conducted in Ethiopia, as evidenced by the emergency obstetric and newborn care reports of 2016, which indicated a median hospital stay of 3 days (30).

However, the result was lower than the mean length of hospital stay following cesarean reported in other countries, such as Australia 6.21 days (8), Nepal 7 days (32), India 8.6 days (27), North-eastern Italy 4.7 days (5), and a median of 5.9 (±3.4) days in 30 low- and middle-income countries (23). This research found that the country's inadequate maternity and neonatal healthcare, a high patient flow rate, limited resources, early discharge preferences, and other factors might be responsible for the variations.

The women in the age group of 20–24 years were five times more likely to be discharged from the hospital early compared to those aged 35 years or older. However, the finding of this study was inconsistent with studies conducted in 30 low- and mid-income countries showed that fewer hospitalization days were associated with higher age ranges >35 years old (23). This could be due to younger women prefer shorter hospital stays due to their better overall health, lower risk of complications, improved physical fitness, fewer comorbidities, and superior nutritional status (33).

Similarly, women who traveled to a hospital for 30–60 min, as compared to those who traveled for more than 60 min, were 2.5 times more likely to be discharged early from the hospital than those who were discharged at the appropriate time. However, the results of this study were inconsistent with a study conducted in Nepal, where a travel time to the health facility of less than 60 min was associated with a longer hospital stay (32). This might be because women living closer to the hospital benefit from shorter travel, reduced costs, better access to care, and greater comfort in their home environment.

The women whose monthly income was less than 2,000 ETB compared to those whose monthly income was greater than or equal to 4,000 ETB were 3 times more likely to be discharged early from the hospital than those who were discharged on appropriate days. This result was inconsistent with a study done in Canada where women with low income were at risk of prolonged hospital stay (33). This may be attributed to financial constraints, the desire to avoid additional financial burdens, a lack of social support, limited access to healthcare, and the need to return to work.

However, women who paid hospital costs with health insurance, in comparison to those who self-paid, were 65% less likely to experience shorter stays or early discharge from the hospital than those who were discharged at the appropriate time. This result is supported by the study conducted in the US and California (32, 34). This might be because health insurance can reduce financial constraints, enabling individuals to receive all necessary treatments and care for their recovery. Consequently, they are less likely to face difficulties staying in the hospital and are less exposed to hospital costs or medication-related expenses.

Similarly, Women who booked their first antenatal care (ANC) within the first trimester (0–13 weeks), as contrasting to those who booked their last trimester (after 28 weeks), were 84.0% less likely to experience shorter stays or early discharge from the hospital compared to those who were discharged at the appropriate time. This finding contradicts the results of a study conducted in rural areas of India on the same population (28). This might be due to this study being conducted in an institution-based study and those women who utilized earlier antenatal care had adequate information about their health status, received timely and comprehensive prenatal care, more likely to be connected with the healthcare providers and facilities that can provide postnatal care and support after childbirth.

Furthermore, women who received counseling and advice about their health status and post-operative care before being discharged from the hospital, as compared to those who did not receive counseling and advice, were 91.0% less likely to be discharged early than those who were appropriately discharged. This is justified as women who receive counseling feel more confident in their ability to manage their recovery, recognize potential complications, and are more likely to adhere to their recovery plan, which can help reduce anxiety, promote better self-care, and improve communication with healthcare providers.

On the other hand, this study showed that the women who developed postoperative complications were 3.5 times more likely to have longer hospital stays than those who were discharged on appropriate days, in comparison to those who did not experience postoperative complications. Several studies conducted in 30 low- and middle-income countries, as well as in Scotland, North-eastern Italy, and the Netherlands, support the finding that hospital stays are longer for women who develop postoperative complications. Antenatal and obstetric complications play a significant role in determining the length of stay following specific practice patterns (5, 23, 35, 36). The possible explanation could be that they may require additional medical interventions, experience delayed recovery, face a higher risk of infection, and need extra psychosocial support and blood transfusions, leading to an extended hospital stay.

Also, those women who gave birth to multiple births compared to women who gave birth to singleton neonates were 2.5 times more likely to stay longer than those who were discharged at the appropriate time from hospitals. This result is supported by a study conducted in northeastern Italy (5). This might be due to women who have multiple births face a higher risk of complications, including prematurity and low birth weights, which necessitate more medical attention and extensive neonatal care, such as admission to the NICU.

Furthermore, women whose Cesarean was performed due to severe pre-eclampsia were 2.8 times more likely to have extended hospital stays compared to those who had cesarean for non-reassuring fetal heart rate patterns. The same finding was reported by different studies in Brazil and Scotland, indicating that women with hypertensive disorders of pregnancy had longer hospital stays after cesarean (30, 35). The possible explanation could be women with pre-eclampsia require longer hospitalization for careful blood pressure monitoring, treatment completion, and ensuring the health of both the mother and the neonate.

In this study, the place of delivery was not associated with the length of stay. This may be due to national norms and the healthcare system affecting the length of stay after birth.

### 4.1 Limitations of the study

This study has several limitations. First, while the length of hospital stay is an important outcome measure, it may not fully reflect the health outcomes of mothers and infants after discharge. Second, the study was conducted exclusively in public hospitals, which could restrict the generalizability of the findings to other settings. To enhance the robustness of future research, it would be advisable to incorporate qualitative techniques and broaden the range of study settings. Finally, it is important to note the lack of sufficient literature on the topic.

## 5 Conclusion

This study found that the length of hospital stay was less than the mean days of hospital stay recommended by the Ethiopian Ministry of Health. Women aged 20–24 years, distance from hospitals 30–60 min, first ANC booking, monthly income less than 2,000 ETB, and women who had health insurance and were counseled before discharge were factors associated with the shorter stay while, women with postoperative complications, severe pre-eclampsia, and multiple births were factors associated with extended hospital stay.

### 5.1 Recommendation

Enhance Accessibility to Antenatal Care: Promote early antenatal booking to reduce the length of hospital stays by ensuring timely management of pregnancy-related issues and complications. This could involve community outreach programs, education campaigns, and improving healthcare infrastructure in rural areas.

Improve Counseling Services: Strengthen counseling services for pregnant women, emphasizing the importance of antenatal care, birth preparedness, and early recognition of danger signs. This could be achieved through training healthcare providers in effective counseling techniques and allocating resources for comprehensive maternal health education programs.

Address Socioeconomic Barriers: Implement measures to address socioeconomic disparities affecting maternal healthcare utilization, such as providing financial assistance or subsidies for low-income families and expanding health insurance coverage to improve access to timely and quality care.

Enhance Postoperative Care Protocols: Develop and implement standardized protocols for postoperative care following cesarean deliveries, focusing on early detection and management of complications to prevent prolonged hospital stays. This may involve training healthcare personnel, improving facilities, and ensuring access to essential medications and equipment.

Multidisciplinary Approach to Complication Management: Foster collaboration between obstetricians, neonatologists, and other relevant healthcare professionals to provide comprehensive care for women with high-risk pregnancies or postoperative complications. This may include regular case reviews, interdisciplinary consultations, and coordinated care plans tailored to individual patient needs.

## Data Availability

The raw data supporting the conclusions of this article will be made available by the authors, without undue reservation.

## Footnotes

^1^Ethiopia Ministry of Health (MOH). Obstetrics Management Protocol for Hospitals. Ethiopia (2021). 219.

^2^Ethiopian Public Health Institute, Federal Ministry of Health, Columbia University. Ethiopian Emergency Obstetric and Newborn Care (EmONC) Assessment. Ethiopia (2016).
